# Tumor Hypoxia Drives Genomic Instability

**DOI:** 10.3389/fcell.2021.626229

**Published:** 2021-03-16

**Authors:** Ming Tang, Emma Bolderson, Kenneth J. O’Byrne, Derek J. Richard

**Affiliations:** ^1^Centre for Genomics and Personalised Health, Queensland University of Technology (QUT), Brisbane, QLD, Australia; ^2^Cancer and Ageing Research Program, Translational Research Institute, Brisbane, QLD, Australia; ^3^Princess Alexandra Hospital, Brisbane, QLD, Australia

**Keywords:** genomic instability, tumor hypoxia, DNA damage repair, DNA damage response, conceptual lethality, HIF-1α, cancer therapeutic resistance

## Abstract

Cancer is a leading cause of death worldwide. As a common characteristic of cancer, hypoxia is associated with poor prognosis due to enhanced tumor malignancy and therapeutic resistance. The enhanced tumor aggressiveness stems at least partially from hypoxia-induced genomic instability. Therefore, a clear understanding of how tumor hypoxia induces genomic instability is crucial for the improvement of cancer therapeutics. This review summarizes recent developments highlighting the association of tumor hypoxia with genomic instability and the mechanisms by which tumor hypoxia drives genomic instability, followed by how hypoxic tumors can be specifically targeted to maximize efficacy.

## Introduction

Hypoxia is a common characteristic of solid tumors (Bristow and Hill, 2008). It arises in tissues when oxygen consumption by the cells outpaces supply, due to elevated oxygen demand in metabolically active cells and decreased oxygen transport to the center of the tumor because of inefficient vascularization. Hypoxia plays an important role in regulating the 11 cancer hallmarks, including metabolic reprogramming, genomic instability, alternative splicing, etc. (Farina et al., 2020). Transcription factors called hypoxia-inducible factors (HIFs) play a significant role in the cellular responses to hypoxia, through the increased transcription of proteins involved in pathways such as apoptosis, proliferation, migration, metabolism, and DNA damage response (Muz et al., 2015).

Genetic instability is a universal hall mark of cancers. In cancer, genetic instability may be seen in different forms, including microsatellite instability, elevated frequencies of base-pair mutations, and chromosomal instability (Negrini et al., 2010). As a major form of genomic instability, chromosomal instability comprises aberrant chromosome numbers (i.e., aneuploidy or polyploidy) and structural changes in chromosomes. The structural chromosome alterations may arise at the chromosome level (e.g., translocations and gains or losses of large portions of chromosomes) or at the nucleotide level, which influence gene structure or expression such as mutations, insertions, deletions, gene amplifications, and gene silencing by epigenetic effects (Jefford and Irminger-Finger, 2006). These changes and markers of genetic instability are driven by a failure of DNA repair systems and cell cycle regulation. Genomic instability is characterized by an elevated propensity of alterations in the genome throughout the cell cycle, where coordinated cell cycle progression and error-free repair of DNA damage are crucial for maintaining genomic integrity. Hypoxia has been demonstrated to impair the tumor cells’ capabilities to maintain genetic integrity, subsequently resulting in loss of coding sequence and genomic instability (Luoto et al., 2013).

## Tumor Hypoxia Correlates With Genomic Instability

There is a clear consensus in studies in which tumor genomes have been sequenced and analyzed that hypoxia is associated with tumor genomic instability. Specifically, hypoxia was found to correlate with significantly elevated genomic instability in 10 tumor types (Bhandari et al., 2019). For example, hypoxic tumors exhibited genomic instability as reflected by elevated rates of chromothripsis, allelic loss of Phosphatase and Tensin Homolog (PTEN) and shorter telomeres in localized prostate cancers (Bhandari et al., 2019). Likewise, hypoxic breast cancer cells showed higher propensity to increase expression of oncogenes and to decrease expression of tumor suppressor genes (Jefford and Irminger-Finger, 2006). Furthermore, in a further study, Bhandari et al. (2020) found that hypoxia and genomic instability correlate across 50% of 1,188 tumors, spanning 27 cancer types, which may synergistically portend rapid relapse for cancer patients after primary treatment (Lalonde et al., 2017). This study found that higher level of hypoxia is associated with elevated mutational burden, and that tumor hypoxia correlates with elevated incidence of mutations in oncogenes and tumor suppressors genes (Bhandari et al., 2020). Additionally, hypoxia was found to link with mitochondrial genome mutations (Hopkins et al., 2017) and centrosome aberrations in cancers (Mittal et al., 2017), both of which correlate with poor prognosis.

Adaptation of cancer cells to hypoxia facilitates tumor aggressiveness *via* driving genomic instability (Reynolds et al., 1996), tumor progression, and therapeutic resistance, thus resulting in poor prognosis (Luoto et al., 2013; Zhang et al., 2020). Indeed, a recent study revealed that hypoxia increases the mutational burden of breast cancer cells, as reflected by elevated frameshift insertions and deletions (Hassan Venkatesh et al., 2020). How hypoxia drives genomic instability is discussed in more detail below.

## Tumor Hypoxia Increases Replication Stress, Induces Alternative Splicing, and Activates DNA Damage Response

In response to DNA damage, cells initiate complex DNA damage response (DDR) pathways, including the phosphorylation of multiple repair proteins by the effector kinases DNA-Dependent Kinase (DNA-PK), Ataxia Telangiectasia and RAD3-related (ATR), and Ataxia Telangiectasia Mutated (ATM) (Richard et al., 2008). Hypoxia has been shown to indirectly cause DNA damage in the form of replication stress, as evidenced by ATR-dependent, hypoxia-induced accumulation of gamma-H2AX (Economopoulou et al., 2009). In addition to the hypoxia-induced response of ATR, ATM has also been shown to have a protective role in the cellular response to hypoxia. As such, depletion of ATM in hypoxic cells led to an increase in DNA damage and reduced rates of replication, supporting a role of ATM in prevention of hypoxia-induced damage and maintenance of replication fork integrity under hypoxic conditions (Olcina et al., 2013).

Oxygen is an important cofactor for mammalian ribonucleotide reductase. Ribonucleotide reductase, comprising ribonucleoside-diphosphate reductase subunit M1/ribonucleoside-diphosphate reductase subunit M2 (RRM1/RRM2) and ribonucleoside-diphosphate reductase subunit M1/ribonucleoside-diphosphate reductase subunit M2 B (RRM1/RRM2B), represents the only enzyme that is capable of *de novo* synthesis of deoxyribonucleotide triphosphates (dNTPs), which are the building blocks of DNA. Under hypoxia, ribonucleotide reductase switches from RRM1/RRM2 to RRM1/RRM2B, due to the fact that RRM1/RRM2B is capable of synthesizing dNTPs under hypoxic conditions, although at a much lower rate. This shift keeps DNA replication active in hypoxic cells, which helps in cellular activities like proliferation (Foskolou et al., 2017). As mentioned above, RRM1/RRM2B synthesizes dNTPs at a much lower level under hypoxia than RRM1/RRM2 in normoxia [as displayed in the Graphical Abstract of Ref. (Foskolou et al., 2017)], which induces replication stress, one of the major sources of genomic instability in cancer, and subsequently activates the DDR (Ng et al., 2018; Olcina et al., 2013). Moreover, acute intermittent hypoxia has been reported to upregulate reactive oxygen species (ROS) (Hassan Venkatesh et al., 2020; Wang et al., 2019) and induces replication stress in various cancers (Pires et al., 2010). Similarly, hypoxia was found to increase replication stress (Hassan Venkatesh et al., 2020) in breast cancer cells, which subsequently activates the DDR, including ATR-, DNA- PK-, and ATM- mediated signaling (Ng et al., 2018; Olcina et al., 2013). Additionally, hypoxia stimulates mitochondria to produce large amount of ROS (Peers et al., 2007), which in turn stabilizes Hypoxia-Inducible Factor 1 alpha (HIF-1α), further inducing replication stress and genomic instability (Chandel et al., 1998).

Another driving force of genomic instability is the hypoxia-induced alternative splicing of DDR and DNA repair genes (Salas-Armenteros et al., 2019). For example, hypoxic colorectal cancer cells shift the DDR pathway coding transcripts to non-coding intron-retained alternative spliced transcripts in the HDAC6 gene encoding histone deacetylase 6, which leads to impaired DNA double-strand break (DSB) repair and genomic instability, by modulating the inhibitory alternative splicing of the tumor suppressor p53-binding protein 1 (TP53BP1) and TP53 co-factor (Memon et al., 2016). Additionally, hypoxia downregulates the DDR by inactivating DDR genes (e.g., TP53), *via* promoting alternative intronic retention splicing and splice-dependent intron-retention nonsense mediated decay (Han et al., 2017).

Some other studies suggested that hypoxia alone may not induce DNA damage that is detectable by the lack of TP53BP1 foci and no increase in Olive tail lengths in alkaline comet assays carried out on hypoxic cells (Hammond et al., 2003; Hassan Venkatesh et al., 2020). However, it can also be hypothesized that hypoxia may induce DNA damage at levels below our current levels of experimental detection. For example, the lack of 53BP1 foci may be due to hypoxia-induced transcriptional and alternative splicing of TP53BP1 (Memon et al., 2016). Similarly, the lack of increase in Olive tails in alkaline comet assays may be caused by reduced conversion of oxidized bases to DNA strand discontinuities, due to the hypoxia-induced downregulation of OGG1, which functions as initiating repair of the major oxidized base, 8-oxoguanine in the context of hypoxia-induced ROS burst (Chan et al., 2014). Downregulation of OGG1 may prevent or delay the excision of damaged bases and the formation of DNA SSBs during the base excision repair process, and consequently this DNA damage would not be detected *via* an alkaline comet assay. Direct detection of 8-oxoguanine using a specific antibody against this form of DNA damage may address this further. As compared to hypoxia, reoxygenation upregulates ROS in a much higher rate and therefore induces a significant increase in DNA damage in cancer cells (Wang et al., 2019).

## Tumor Hypoxia Alters DNA Repair Pathways

Genomic instability is highly fostered by alterations in DNA repair pathways. Indeed, numerous studies revealed that hypoxia alters many DNA repair pathways, including homologous recombination repair (HRR), non-homologous end-joining (NHEJ), microhomology-mediated alternative end joining (MMEJ), mismatch repair (MMR), nucleotide excision repair (NER), base excision repair (BER), translesion DNA synthesis (TLS), and Fanconi Anemia (FA)-mediated DNA repair. The hypoxia-induced changes in DNA repair pathways can originate either from genetic alterations in the expression and signaling of DNA repair proteins or from modifications in epigenetic enzymes [e.g., α-Ketoglutarate (KG)-dependent histone lysine demethylase (KDM) and ten eleven translocation (TET) DNA demethylases] functioning in histone modification at the chromatin level and DNA methylation at the DNA level (Begg and Tavassoli, 2020; Kaplan and Glazer, 2020; Luoto et al., 2013; Meng et al., 2005; Scanlon and Glazer, 2015). Specifically, hypoxic exposure downregulates genes involved in DNA replication and repair pathways, thus increasing genomic instability (Hassan Venkatesh et al., 2020). Interestingly, tumor hypoxia can epigenetically regulate DNA DSB repair by inducing the accumulation of oncometabolites, such as succinate, fumarate, 2-hydroxyglutarate (2-HG) and D-2-hydroxyglutarate, which are competitive inhibitors of the αKG-dependent KDM and TET families of epigenetic enzymes that function in DSB repair pathways like HRR and NHEJ (Xiang et al., 2020).

Hypoxia-mediated suppression of DNA repair pathways were previously reviewed in Meng et al. (2005), Luoto et al. (2013), Scanlon and Glazer (2015), Begg and Tavassoli (2020), Kaplan and Glazer (2020), and Xiang et al. (2020). The following sections highlight recent developments on the hypoxia-induced alterations in DNA repair proficiency.

### Hypoxia Alters DNA Double-Strand Break Repair

Double-strand breaks represent the most lethal form of DNA damage. Failure in repair of damaged DSBs can result in chromosomal instability such as aneuploidy, deletions (loss of heterozygosity), and chromosomal translocations (Hoeijmakers, 2001). There are three main DSB repair mechanisms: HRR, NHEJ, and MMEJ. Among the three, HRR is the most high-fidelity pathway that repairs DSBs by utilizing a homologous sequence as a template. It is well established that hypoxia downregulates the expression of HRR genes *via* transcriptional, translational, and epigenetic repression, which results in reduced HRR capacity and consequently causes mutagenesis and promotes genomic instability (Kaplan and Glazer, 2020; Meng et al., 2005; Scanlon and Glazer, 2015). For example, hypoxia was reported to induce the downregulation of DNA DSB repair genes, including HRR genes such as RAD51, RAD51B/C/D, RAD54B, and XRCC2/3 (Meng et al., 2005). The hypoxia-induced downregulation of several HRR genes (e.g., BRCA1 and RAD51) were found to be mediated by transcriptional repressors E2F4/P130 involved in the E2F pathways, which is independent of the expression of HIF-1α and cell cycle phase (Scanlon and Glazer, 2015). Furthermore, hypoxia has been recently found to correlate with defective HRR in tumors, as suggested by whole-genome sequencing analysis of 1,188 tumors across 27 cancer types (Bhandari et al., 2020). Additionally, hypoxia was found to induce mutations in numerous HRR genes, for example, Breast Cancer Associated 1 and 2 (BRCA1 and BRCA2) and to downregulate HRR gene expression (e.g., RAD52), thus impairing HRR (Hassan Venkatesh et al., 2020). Interestingly, hypoxia also inhibits deubiquitylating enzyme ubiquitin-specific peptidase 11 (USP11) (Martín Mateos, 2019), depletion of which causes HRR deficiency in cancer cells (Wiltshire et al., 2010).

Whilst hypoxia clearly downregulates HRR, conflicted effects of hypoxia on the more error-prone NHEJ pathway have been reported (Kaplan and Glazer, 2020; Scanlon and Glazer, 2015). This discrepancy may arise from the difference in cancer types and hypoxic conditions. However, recent examination on the expression of 180 DNA repair related genes in various cancer cell lines found that hypoxia downregulated most NHEJ genes (Cowman et al., 2020). Notably, the expression of DNA Ligase IV (LIGIV), a key component of NHEJ, was downregulated more than 1.5-fold by hypoxia, which agrees with previous findings for prostate cancers (Meng et al., 2005). Similarly, hypoxia caused downregulation (Meng et al., 2005) and mutation (Hassan Venkatesh et al., 2020) of the XRCC4 gene in different cancer cell lines, thus compromising NHEJ (Sulkowski et al., 2018). Interestingly, hypoxia suppresses the expression of the tumor suppressor PTEN in tumors (Bhandari et al., 2020), which leads to a deficiency in NHEJ-mediated DSB repair (Sulkowski et al., 2018).

The effect of hypoxia on another DSB repair mechanism, MMEJ, is not clear. Similar to NHEJ, the influence of hypoxia on MMEJ was found conflicted for different cancer types and for different hypoxic conditions. For example, chronic hypoxia downregulated and intermittent hypoxia upregulated the MMEJ essential gene, FEN1 expression in MCF-7 cells, whereas intermittent hypoxia downregulated FEN1 in MDA-MB-231 cells (Hassan Venkatesh et al., 2020; Sharma et al., 2015).

In summary, tumor hypoxia either downregulates DSB repair pathways or induces a switch from the high-fidelity HRR to more error-prone NHEJ and MMEJ, both of which are more likely to induce structural chromosomal instability, such as insertions and deletions in nucleotide, thus driving genomic instability. For example, hypoxia transcriptionally and translationally decreased BRCA1, thus downregulating the HRR pathway (Bindra et al., 2005). However, NHEJ pathways remained active, suggesting a hypoxia-induced switch of DNA repair from HRR to NHEJ, which may contribute to genomic instability (Bindra et al., 2005).

### Hypoxia Downregulates Mismatch Repair

Mismatch repair, which repairs erroneous bases during DNA replication and recombination, is important for genomic integrity. Cells with deficiency in MMR grow with high mutational rates, develop a mutator phenotype and induce microsatellite instability, thus causing the accumulation of genomic alterations (Scanlon and Glazer, 2015). It is well recognized that genes central to efficient MMR (e.g., MLH1, MSH2, and MSH6) are transcriptionally, translationally, and epigenetically downregulated by hypoxia, thus increasing mutagenesis and microsatellite instability (Kaplan and Glazer, 2020; Koshiji et al., 2005; Lu et al., 2014; Mihaylova et al., 2003; Scanlon and Glazer, 2015). For example, hypoxia was demonstrated to reduce histone H3 lysine 4 (H3K4) methylation at the MLH1 promoter and resulting in epigenetically driven silencing of MLH1, which is a common phenomenon in sporadic colorectal cancers (Lu et al., 2014). This silencing of gene MLH1 may induce structural chromosomal alterations at the nucleotide level (Lu et al., 2014). Interestingly, the transcriptional downregulation of MMR genes MLH1, MSH2, and MSH6 are all dependent on the signaling pathways involving transcriptional factor HIF-1α (Scanlon and Glazer, 2015).

It has been recently found that defective MMR is closely related to elevated hypoxia in tumors, based on the results from whole-genome sequencing analysis of 1,188 tumors spanning 27 tumor types (Bhandari et al., 2020). Additionally, hypoxia was recently found to decrease PMS2, a gene essential for MMR, through a HIF-1α-independent manner (Cowman et al., 2020), which is consistent with previous findings (Begg and Tavassoli, 2020; Mihaylova et al., 2003). This study also revealed that hypoxia drastically downregulates MSH2 in different brain cancer cell lines (Cowman et al., 2020). Interestingly, MSH2 and MSH3 were found to be mutated in breast cancer cells under hypoxic conditions (Hassan Venkatesh et al., 2020). The role of these mutations in regulating the MMR capacity of hypoxic tumor cells is so far not clear. For cells with defective MMR capacity, mutations in MMR genes may restore the MMR capacity if such a mutation restores transcription.

### Hypoxia Downregulates Base Excision Repair

Base excision repair is the major mechanism used by cells to repair oxidized DNA lesions. Hypoxia can lead to the downregulation of BER, which potentially increases the frequency of base-pair mutations and drives genomic instability. For instance, chronic hypoxia significantly downregulated many BER repair proteins in cancer cells such as OGG1, MYH, APE1, and MTH1 (Chan et al., 2014), impairing BER-mediated repair, thus causing the accumulation of residual base damage and driving genomic instability. Similarly, hypoxia suppresses the expression of zinc-finger protein ATMIN, which is involved in BER (Jurado et al., 2010), through transcription factors p53 and HIF-1α in an oxygen dependent manner (Leszczynska et al., 2016). Additionally, hypoxia may impair the function of BER pathways (Pennaneach and Hall, 2017), by preventing the accumulation of XRCC1 at DNA SSBs (Ahmed, 2017) or by inducing somatic mutations in the XRCC1 gene (Hassan Venkatesh et al., 2020).

### Hypoxia Promotes Translesion DNA Synthesis

Translesion DNA synthesis is a process adopted by cells where specialized polymerases bypass DNA lesions to avoid replication fork stalling (Goodman and Woodgate, 2013). This lesion-tolerance DNA repair pathway is error-prone and often associated with genomic instability. The exact impact of hypoxia on TLS is not yet clear. However, evidence indirectly indicates that hypoxia might increase the propensity of TLS used by tumor cells to repair DNA damage, thus promoting the accumulation of genomic instability. For instance, a recent study suggested that hypoxia may facilitate error-prone TLS in prostate cancer, thus driving tumor genomic instability (Luo et al., 2020). This study found that hypoxia remarkably downregulated SDE2, a DNA replication stress mediator, and activates TLS. Depletion of SDE2 enhanced cellular sensitivity to DNA damage and suppressed tumor growth, suggesting that SDE2 may be a potential therapeutic target for hypoxic tumors (Luo et al., 2020).

### Hypoxia Downregulates Fanconi Anemia-Mediated DNA Repair

The FA repair pathway is essential for the repair of DNA interstrand crosslinks (ICLs) (Longerich et al., 2014; Walden and Deans, 2014). In response to ICLs, the FA core complex is recruited to the resulting stalled replication forks and monoubiquitinates another two FA proteins FANCD2 and FANCI, thus initiating an ICL repair response including TLS and re-establishment of replication forks by HR (Walden and Deans, 2014).

Chronic hypoxia was found to downregulate FANCD2 in cancer cells independent of the HIF-1α expression, resulting in an impaired FA pathway, contributing to genomic instability (Scanlon and Glazer, 2014). Similarly, hypoxia downregulated the expression of UBE2T, a ubiquitin ligase required for FADNA repair, in several cancers in a HIF-1α-independent manner, thus enhancing their sensitivity to ICL-inducing agents such as mitomycin C (MMC) (Ramaekers et al., 2011). Additionally, hypoxia significantly decreases excision repair cross complementation group 1 protein (ERCC1) (Dudás et al., 2014), which plays an essential role in FA for DNA ICL repair (Douwel et al., 2014). Notably, hypoxia was found to induce mutations in numerous FA genes such as FANCI in breast cancer cells (Hassan Venkatesh et al., 2020). Collectively, these results suggest that the FA pathway can be targeted to treat hypoxic tumors.

## Tumor Hypoxia Can Promote Cell Proliferation and Enhance Therapeutic Resistance

Cancer is characterized by uncontrollable cell proliferation. During a certain period of time, the higher the proliferation rate is, the more likely cells can acquire genomic changes, including antimorphic mutations for oncogenic activation and amorphic mutations in tumor suppressor genes that may further promote proliferation. Under hypoxia, tumors drive genomic instability partially by promoting uncontrollable cell proliferation. This can be achieved by hypoxia-induced dysregulation of cell cycle checkpoints *via* enhancing oncogene (e.g., MYC) expressions (Gordan et al., 2007) and suppressing tumor suppressors (e.g., PTEN) (Bhandari et al., 2020), or by dysregulating the expression of proteins involved in cell cycles and metabolism. For instance, hypoxia upregulates the secretion and expression of fractalkine (FKN), thus enhancing the proliferation rates by promoting the G1/S phase transition in prostate cancers (Tang et al., 2015). Additionally, hypoxia significantly upregulates the metabolic driver squalene monooxygenase (SQLE), thus promoting cell proliferation in hypoxic tumors (Haider et al., 2016; Sui et al., 2015).

**FIGURE 1:**
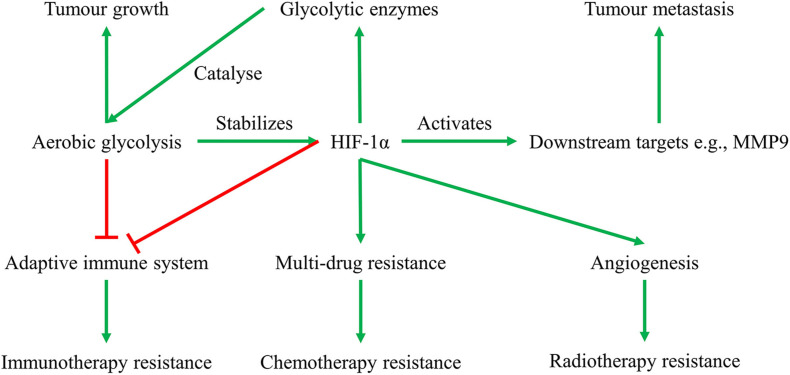
Feed-forward loop of glycolysis-HIF-1α signaling pathway, and their roles in solid tumor growth, metastasis, and therapeutic resistance. Glycolysis provides energy and nutrients for tumor growth, suppresses anti-tumor T-cells causing immunotherapy resistance, and enhances HIF-1α activities. HIF-1α in turn transactivates glycolytic enzymes that catalyze glycolysis, activates downstream signaling pathways facilitating tumor metastasis, and induces tumor therapeutic resistance in radiotherapy, chemotherapy, and immunotherapy.

Another significant approach by which hypoxic tumors maintain continuous rapid cell proliferation is cellular metabolic reprogramming. Among the changes in tumor cellular metabolism, elevated glycolysis is one of the most striking (Hanahan and Weinberg, 2011). Glycolysis is defined as a sequence of 10 enzyme-catalyzed reactions that convert glucose into pyruvate and release energy in the form of adenosine triphosphate (ATP) (Yu et al., 2017). In healthy cells, pyruvate then enters the mitochondrial tricarboxylic acid (TCA) cycle in normoxia (3–15% O_2_) when there is abundant oxygen and produces 36 ATP, or is converted to lactate in low-oxygen condition (<1% O_2_) termed “hypoxia” and releases only two ATP (Yu et al., 2017). Unlike normal cells that only prioritize glycolysis in hypoxia, tumor cells prefer to produce energy through glycolysis even in normoxia. Therefore, tumor glycolysis is often defined as “aerobic glycolysis” or the Warburg effect (Warburg, 1956), to be different from the anaerobic glycolysis of healthy cells. Glycolysis is essential for providing tumor cells with energy and nutrients, thus facilitating their uncontrollable proliferation (Garber, 2006). Due to the low efficiency of glycolysis in producing energy (two ATP molecules per glucose compared versus 36 ATP molecules per glucose for oxidative phosphorylation), cancer cells uptake and metabolize more glucose. This is achieved by activating HIF-1 to transactivate several hundred target genes (Samanta and Semenza, 2018). HIF-1 consists of an oxygen-dependent α subunit and an oxygen-independent β subunit. The α subunit has three isoforms, that is, HIF-1α, HIF-2α, and HIF-3α, which is stabilized and transported into the nucleus and dimerizes with a β subunit when oxygen concentrations is below 6% (Schofield and Ratcliffe, 2004). This heterodimer binds to the core sequence 5-RCGTG-3 of hypoxia responsive element (HRE) within the enhancer promoter region of numerous HIF-1 target survival genes. Consequently, HREs facilitate the transcription of target survival genes including those encode glucose transporters (Wood et al., 2007) and glycolytic enzymes (Iyer et al., 1998; Marin-Hernandez et al., 2009). In addition to upregulating the expression of glucose transporters and glycolytic enzymes required for glycolysis, HIF-1 inhibits mitochondrial oxidative phosphorylation by blocking pyruvate entry and the conversion of pyruvate to acetyl-CoA (Zeng et al., 2015), thus further stimulating glycolysis and promoting tumor cell survival even in acute and prolonged hypoxic conditions. Interestingly, the HIF-1α-promoted aerobic glycolysis in turn stabilizes HIF-1α, forming a feed-forward loop that promotes tumor growth (Figure 1) (Grandjean et al., 2016). Apart from functioning to regulate energy production pathways, HIF-1α also reduces cancer patient survival by: (1) upregulating many survival proteins such as vascular endothelial growth factor (VEGF) for vascularization and angiogenesis, causing radiotherapy resistance (Kelly et al., 2003); (2) inhibiting cells’ adaptive immune system, resulting in immunotherapy resistance (Hatfield and Sitkovsky, 2016); (3) facilitating cellular apoptosis resistance, leading to multi-drug resistance in chemotherapy (Favaro et al., 2008); and (4) activating its downstream signaling targets, for example, MMP9, thus stimulating tumor metastasis (Chang et al., 2017) and inducing a poor prognosis (Swinson et al., 2004) (Figure 1). Indeed, elevated levels of HIF-1α have been found to be associated with poor patient survival rates in 19 types of cancers (Zhong et al., 1999). Collectively, HIF-1α is a promising drug target for anticancer therapies (Seeber et al., 2010).

In addition to transcriptional adaptation, hypoxia triggers extensive post-transcriptional events for cell proliferation *via* regulation of mRNA stability, altering alternative splicing decisions and translation efficiency. For example, hypoxia specifically induces Muscleblind-Like Protein 2 (MBNL2), therefore enhancing tumor cell proliferation (Fischer et al., 2020). This is achieved by controlling the mRNA levels of HIFs target genes or by regulating widespread hypoxia-dependent alternative splicing, some of which can cause genomic instability (Teye et al., 2017).

## Tumor Hypoxia Facilitates Cellular Apoptosis Resistance

Tumor cells behave differently under different hypoxic conditions. In response to severe and prolonged hypoxia, tumor cells undergo programmed cell death facilitated by HIF-1α, which stabilizes the apoptotic protein p53 and inhibits the antiapoptotic activity of B-cell lymphoma 2 (Bcl-2). However, under acute and mild hypoxia, cancer cells can adapt to the hypoxic stress and continue to grow into a more aggressive phenotype (Greijer and Van der Wall, 2004). Specifically, hypoxia induces transcription factor Nuclear Factor κB (NF-κB), which upregulates the inhibitor of apoptosis protein 2 (IAP-2), thus causing cellular apoptosis resistance. Additionally, hypoxia stabilizes HIF-1α which may possess an antiapoptotic function, as tumor cells with elevated HIF-1α show higher resistance to apoptosis.

Hypoxia induces resistance to apoptosis in cancer cells by increasing the expression of anti-apoptotic proteins or by decreasing the expression of apoptosis proteins. For instance, tumors with mutations in the anti-apoptotic gene BCL2 possess lower levels of hypoxia than those without (Bhandari et al., 2020). Likewise, tumors with mutations in the apoptosis tumor suppressor TP53 showed higher levels of hypoxia in numerous cancer types compared to those without (Bhandari et al., 2020). This agrees with previous findings that dynamic cycling of hypoxia can select for apoptosis-deficient tumor cells and those with TP53 mutations (Graeber et al., 1996), thus enhancing capability to withstand apoptotic stimuli (Kamat et al., 2007).

The hypoxia-induced cellular apoptosis resistance enables tumor cells to proliferate constantly even in the context of unrepaired DNA, thus driving genomic instability. Indeed, in response to reoxygenation followed by acute hypoxia, tumor cells without functional p53 escape apoptosis, thus leading to genomic instability (Pires et al., 2010). Further, hypoxia induces RRM1/RRM2B ribonucleotide reductase, which in turn allows tumor cells to continue to proliferate and escape from hypoxic-specific apoptosis (Foskolou et al., 2017). Similarly, hypoxia was found to upregulate WEE1 (Hong et al., 2011), a G2/M checkpoint inhibitory protein kinase identified as one of the five main inhibitors that protected hypoxic myoblasts from apoptosis (Kang et al., 2015). This enhances cellular apoptosis resistance and protects cancers from therapy-induced DNA damage, causing genomic instability and therapeutic resistance (Wang et al., 2020). In addition, tumor cells induce the DNA/RNA helicase Senataxin (SETX) under radiobiological hypoxia, which inhibits cellular apoptosis assisted by the protein kinase R (PKR)-like endoplasmic reticulum kinase (PERK)/activating transcription factor 4 (ATF4) arm of the unfolded protein response (UPR) (Ramachandran et al., 2020). Inhibition of either SETX or PERK reduces hypoxic tumor survival, suggesting that SETX and PERK are potential therapeutic targets for hypoxic tumors.

## Targeting Tumor Hypoxia and Future Directions

**FIGURE 2:**
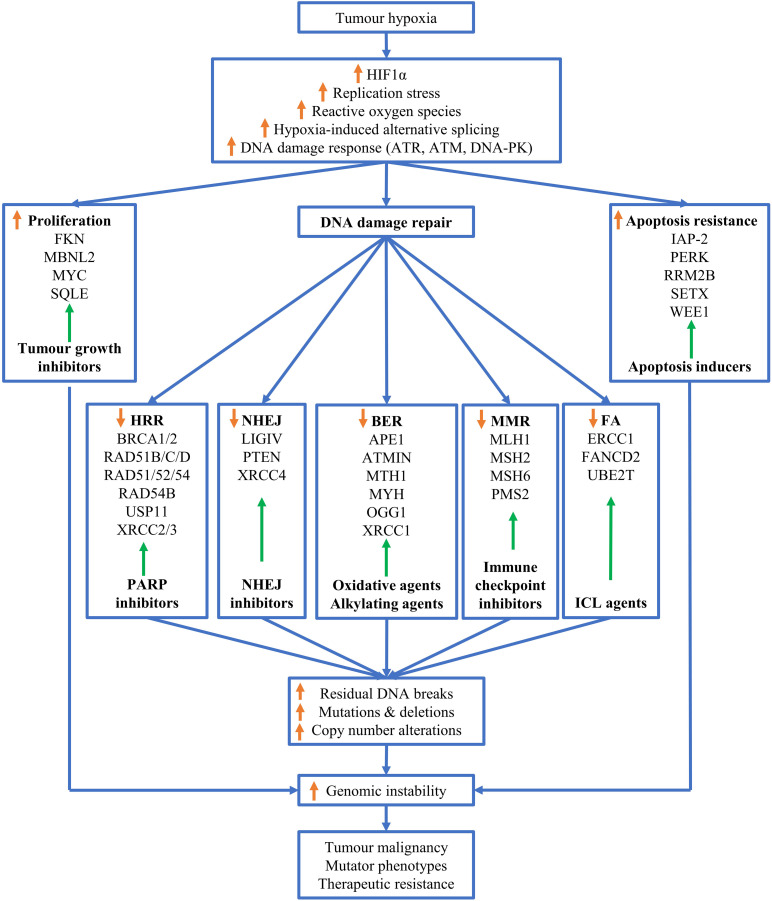
Mechanisms of hypoxia-driven genomic instability. Tumor hypoxia upregulates reactive oxygen species (ROS), replications stress, alternative splicing, stabilizes HIF-1α and triggers the DNA damage response (DDR). These adaptions of tumors to hypoxia promote cell proliferation, facilitate cellular apoptosis resistance and downregulate various DNA repair pathways, thus resulting in increased residual DNA breaks, and mutations and deletions, and copy number alterations. Theses alterations in genomes leads to genome instability, which ultimately drives tumor malignancy, mutator phenotypes and therapeutic resistance. Adaptation to tumor hypoxia can be correspondingly targeted by therapeutic intervention as indicated by green arrows similar to Figure 1 in Ref (Kaplan and Glazer, 2020), for example, hypoxic tumor cells with downregulated homologous recombination repair can be targeted by PARP inhibitors.

Although tumor hypoxia drives genomic instability, it provides valuable therapeutic opportunities for targeting hypoxic tumors (Figure 2). So far, targeting the cellular response to hypoxia has been proven challenging. For example, despite the tremendous efforts devoted into the development of anticancer drugs targeting HIF-1α, none of the inhibitors developed to date has yielded significant clinical effects (Welsh et al., 2004).

Given that hypoxia induces mutations in DNA repair genes and downregulates DNA repair pathways, hypoxic tumors can be alternatively targeted by synthetic lethality and contextual lethality, respectively. Tumors with mutated HR genes, BRCA1 and BRCA2, harbor lower HRR capacity and are more sensitive to Poly (ADP-ribose) polymerase (PARP) inhibitors (PARPi), resulting in synthetic lethality (Rose et al., 2020). Since tumor hypoxia induces mutations in HRR genes, hypoxic cancer cells may be synthetically targeted by PARPi. Additionally, PARPi yielded better effects on hypoxic tumors without mutated DNA repair genes than on normoxia cancer cells, due to the hypoxia-induced downregulation of DNA repair capacities. This effect is termed contextual lethality (Chan and Bristow, 2010). Indeed, hypoxia significantly enhanced the radiosensitivity of head and neck squamous cell carcinoma (HNSCC) cells to the PARPi Olaparib (Lee et al., 2019). It will be interesting for future studies to explore whether hypoxia also imposes lethality to tumor cells harboring mutated HRR genes. It is possible that hypoxia could further downregulate the protein expressed from the remaining HRR genes allele in patients, thus resulting in a contextual loss of heterozygosity, which facilitates tumor aggressiveness. If this is the case, hypoxic tumors with mutated HRR genes would be predicted to be hypersensitive to PARPi, resulting in better patient outcomes.

Urgent research is required to investigate tumor hypoxia and avenues to target hypoxic tumors. Research outcomes into hypoxia control of the DDR have not yet enabled us to optimize treatment plans to improve therapy success rates. Indeed, the hypoxia-activated prodrug Nimorazole adopted in the treatment of HNSCC patients in Denmark is so far the only hypoxia intervention that is incorporated into standard of care. Prodrugs and small molecules that target DDR and genomic instability represent promising agents for the treatment of hypoxic tumors, and hence are worth being explored in future studies.

## Author Contributions

MT wrote the draft and made the figures. DR, EB, and KO’B did thorough review and provided significant comments and revisions. All authors contributed to the article and approved the submitted version.

## Conflict of Interest

The authors declare that the research was conducted in the absence of any commercial or financial relationships that could be construed as a potential conflict of interest.
